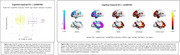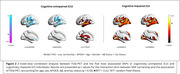# The impact of brain energy metabolism‐related single nucleotide polymorphisms on Alzheimer's disease

**DOI:** 10.1002/alz70856_106392

**Published:** 2026-01-07

**Authors:** Gabriel Lermen Hoffmeister, Christian Limberger, Gabriel Colissi Martins, Mariana Radaelli Schmaedek, Ramon Bertoldi de Souza, Marco Antônio De Bastiani, Eduardo R. Zimmer

**Affiliations:** ^1^ University of Cologne, Cologne, North Rhine‐Westphalia, Germany; ^2^ Universidade Federal do Rio Grande do Sul, Porto Alegre, Rio Grande do Sul, Brazil; ^3^ Universidade Federal do Rio Grande do Sul, Porto Alegre, RS, Brazil; ^4^ Brain Institute of Rio Grande Do Sul, PUCRS, Porto Alegre, RS, Brazil; ^5^ McGill Centre for Studies in Aging, Montreal, QC, Canada

## Abstract

**Background:**

FDG‐PET studies have demonstrated that glucose metabolism abnormalities can be detected up to a decade prior to the clinical onset of Alzheimer's disease (AD). This finding underscores that early metabolic alterations are fundamental to AD pathogenesis, rather than merely secondary to neurodegeneration. While neurons are central to this process, astrocytes also play a pivotal role in maintaining metabolic brain homeostasis. However, the specific contributions of neuron‐astrocyte metabolic interactions to AD pathology remain poorly understood. In this study, we examined the influence of single nucleotide polymorphisms (SNPs) in genes associated with astrocyte‐neuron metabolic cooperation across the AD continuum.

**Method:**

We analyzed genetic variants, FDG‐PET imaging, and cognitive data from 619 cognitively unimpaired (CU) and cognitively impaired (CI) individuals from the ADNI database. An initial screening investigated 3,506 SNPs in genes related to brain energy metabolism. Linear models were used to assess associations between SNP carriership and regional FDG‐PET measures. Voxel‐wise analyses were then conducted for the most significant SNPs, examining their relationship with brain glucose metabolism changes and their potential impact on cognitive decline.

**Results:**

The SNP rs17867763 in the glutamate metabotropic receptor 8 (GRM8) gene showed the strongest association with FDG hypometabolism in cognitively impaired (CI) individuals (Figure 1A). This SNP was also significantly correlated with greater cognitive impairment across six cognitive/clinical assessments, including MMSE and CDR‐SB. Voxel‐wise analysis identified cortical clusters where FDG hypometabolism was linked to cognitive decline (Figure 1B). Additionally, voxel‐wise analysis of five other GRM8 SNPs in both cognitively unimpaired (CU) and CI individuals revealed patterns of FDG hypo‐ and hypermetabolism in brain regions associated with Alzheimer's disease pathology (Figure 2).

**Conclusion:**

The association of GRM8 SNPs with hypo‐ and hypermetabolism supports the hypothesis that synaptic dysfunction and metabolic imbalance contribute to early AD pathology. As mGluR8 modulates synaptic transmission, learning, and memory, its disruption likely affects glucose metabolism and cognitive decline. This highlights the relevance of glutamatergic neurotransmission in supporting optimal brain function in AD.